# BDNF Regulates the Expression and Distribution of Vesicular Glutamate Transporters in Cultured Hippocampal Neurons

**DOI:** 10.1371/journal.pone.0053793

**Published:** 2013-01-11

**Authors:** Carlos V. Melo, Miranda Mele, Michele Curcio, Diogo Comprido, Carla G. Silva, Carlos B. Duarte

**Affiliations:** 1 CNC-Center for Neuroscience and Cell Biology, University of Coimbra, Coimbra, Portugal; 2 Department of Biological and Environmental Science, University of Sannio, Benevento, Italy; 3 Department of Life Sciences, University of Coimbra, Coimbra, Portugal; Baylor College of Medicine, United States of America

## Abstract

BDNF is a pro-survival protein involved in neuronal development and synaptic plasticity. BDNF strengthens excitatory synapses and contributes to LTP, presynaptically, through enhancement of glutamate release, and postsynaptically, via phosphorylation of neurotransmitter receptors, modulation of receptor traffic and activation of the translation machinery. We examined whether BDNF upregulated vesicular glutamate receptor (VGLUT) 1 and 2 expression, which would partly account for the increased glutamate release in LTP. Cultured rat hippocampal neurons were incubated with 100 ng/ml BDNF, for different periods of time, and VGLUT gene and protein expression were assessed by real-time PCR and immunoblotting, respectively. At DIV7, exogenous application of BDNF rapidly increased VGLUT2 mRNA and protein levels, in a dose-dependent manner. VGLUT1 expression also increased but only transiently. However, at DIV14, BDNF stably increased VGLUT1 expression, whilst VGLUT2 levels remained low. Transcription inhibition with actinomycin-D or α-amanitine, and translation inhibition with emetine or anisomycin, fully blocked BDNF-induced VGLUT upregulation. Fluorescence microscopy imaging showed that BDNF stimulation upregulates the number, integrated density and intensity of VGLUT1 and VGLUT2 puncta in neurites of cultured hippocampal neurons (DIV7), indicating that the neurotrophin also affects the subcellular distribution of the transporter in developing neurons. Increased VGLUT1 somatic signals were also found 3 h after stimulation with BDNF, further suggesting an increased de novo transcription and translation. BDNF regulation of VGLUT expression was specifically mediated by BDNF, as no effect was found upon application of IGF-1 or bFGF, which activate other receptor tyrosine kinases. Moreover, inhibition of TrkB receptors with K252a and PLCγ signaling with U-73122 precluded BDNF-induced VGLUT upregulation. Hippocampal neurons express both isoforms during embryonic and neonatal development in contrast to adult tissue expressing only VGLUT1. These results suggest that BDNF regulates VGLUT expression during development and its effect on VGLUT1 may contribute to enhance glutamate release in LTP.

## Introduction

BDNF (brain-derived neurotrophic factor) is a pro-survival protein that promotes neuronal differentiation and synaptic plasticity [1], [2], in addition to neuroprotection [3], [4]. During development, BDNF stimulates the formation of appropriate synaptic connections, controlling the direction and rate of axon growth [5], [6], as well as the shape of dendritic arbors and spines [7]–[9]. In the adult hippocampus, BDNF is also involved in learning [10], [11] and memory formation [12], [13], and is essential for long-term potentiation (LTP) [14]–[18].

The effects of BDNF are mainly mediated through activation of the TrkB (tropomyosin-related kinase B receptor) receptor tyrosine kinase as well as the p75 neutrotrophin receptor (p75^NTR^) [19]. Activation of TrkB receptors by BDNF leads to receptor dimerization and trans-autophosphorylation of several tyrosine residues in the intracellular domain, including Y490 and Y816, which allow recruiting proteins containing PTB and SH2 (Src homology-type 2) domains, activating in parallel the Ras-ERK (extracellular signal-regulated kinase), PI3-K (phosphatidylinositol 3-kinase)/Akt and phospholipase C-γ (PLCγ) signaling pathways [20]. Trans-autophosphorylation of Y816 recruits and activates cytoplasmic PLCγ, which hydrolyzes PIP2 (phosphatidylinositol 4,5-bisphosphate) into IP3 (inositol 1,4,5-trisphosphate) and DAG (diacylglycerol). IP3 promotes Ca^2+^ release from internal stores, activating [Ca^2+^]_i_-regulated enzymes, including Ca^2+^- and calmodulin-dependent protein kinases (CAMKs), and protein kinase C (PKC) isoforms [21]. Concomitantly, DAG stimulates DAG-regulated PKC isoforms, such as PKCδ [20]. The PLCγ pathway is central in LTP [18], [22], [23] and growth cone guidance [6], retrograde synaptic modification [24] and dendritic spine morphology [25] regulated by activation of TRPC (transient receptor potential canonical) channels. Trans-autophosphorylation of Y490 enables recruitment of Shc (Src homology 2-containing protein), IRS1 (insulin receptor substrate 1) and IRS2 linker proteins, thereby activating the Ras-ERK and PI3K/Akt cascades [26]. ERK translocates to the nucleus upon phosphorylation, regulating gene expression through isoform-specific activation of transcription factors, including cAMP-response element binding protein (CREB) (through ERK1/2/5), MEF2 (downstream of ERK5) or Elk1 (following activation of ERK1/2) [27]. The Ras-ERK signaling pathway is crucial for neurogenesis [28], inhibition of proapoptotic proteins [29], stimulation of pro-survival gene expression [30] and protein synthesis-dependent plasticity [31]. The PI3K/Akt pathway has a pivotal role in cell survival [32], neuroprotection [3], trafficking of synaptic proteins [33] and can also directly control protein synthesis through mTOR (mammalian target of rapamycin) activation and 4EBP phosphorylation [34].

The TrkB-activated signaling pathways account for nearly all BDNF synaptic effects but their biological responses likely reflect BDNF or TrkB receptor levels and the spatiotemporal pattern of BDNF stimulation, especially when activated pre- and/or postsynaptically [35]. Nevertheless, the molecular mechanisms underlying BDNF signaling in short-term plasticity and long-term potentiation are not fully understood. We have previously reported that BDNF induces significant proteome changes [36], including the regulation of AMPA and NMDA receptors involved in molecular mechanisms of synaptic plasticity [37], [38]. BDNF promotes phosphorylation of synapsin I [39] and beta-catenin [40] increasing synaptic vesicle docking at the active zone and quantal glutamate release [39], [41]. However, direct presynaptic effectors of protein synthesis-dependent BDNF signaling on glutamatergic function, which also contributes to LTP and memory formation [22], [23], have not been identified thus far. The vesicular glutamate transporters (VGLUT) are such target candidates because they mediate L-glutamate uptake into synaptic vesicles and are required for exocytic glutamate release at presynaptic terminals [42]. Moreover, VGLUT1 and VGLUT2 expression is developmentally regulated in order to match vesicle cycling and quantal amplitude [43], [44]. In addition, VGLUT isoforms have similar substrate specificity, transport activity and kinetics but complementary expression, which correlates with release probability and potential for plasticity [45]. Therefore, the current study aimed at examining the effect of BDNF on the expression of VGLUT, given their relevance in LTP, learning and memory function [46], [47]. We report that BDNF regulates VGLUT gene and protein expression during development of cultured hippocampal neurons, through activation of the PLCγ signaling pathway, and also affects VGLUT subcellular distribution, further suggesting a role in BDNF-induced LTP.

## Materials and Methods

### Ethics Statement

Experiments were performed according to the European Union Directive 86/609/EEC and the legislation Portaria n. 1005/92, issued by the Portuguese Government for the protection of animals used for experimental and other scientific purposes. Dams were sacrificed by cervical dislocation. Embryos were then surgically removed and sacrificed by decapitation.

### Hippocampal Cultures

Primary cultures of rat hippocampal neurons were prepared from the hippocampi of E18–E19 Wistar rat embryos, after treatment with trypsin (0.06%, for 15 min at 37°C; GIBCO-Invitrogen) and deoxyribonuclease I (5.36 mg/ml), in Ca^2+^- and Mg^2+^-free Hank’s balanced salt solution (HBSS; 5.36 mM KCl, 0.44 mM KH_2_PO_4_, 137 mM NaCl, 4.16 mM NaHCO_3_, 0.34 mM Na_2_HPO_4_.2H_2_O, 5 mM glucose, 1 mM sodium pyruvate, 10 mM HEPES and 0.001% phenol red). The hippocampi were then washed with HBSS containing 10% fetal bovine serum (GIBCO-Invitrogen), to stop trypsin activity, and transferred to Neurobasal medium (GIBCO-Invitrogen) supplemented with B27 supplement (1∶50 dilution; GIBCO-Invitrogen), 25 µM glutamate, 0.5 mM glutamine and 0.12 mg/ml gentamycin. The cells were dissociated in this solution and were then plated in 6-well plates (870,000 cells/well) coated with poly-D-lysine (0.1 mg/ml), or on poly-D-lysine coated glass coverslips, at a density of 80,000 cells/well (12-well plates). The cultures were maintained in a humidified incubator of 5% CO_2_/95% air, at 37°C, for 7 or 14 days. BDNF stimulation was carried out by adding BDNF (Regeneron or PeproTech) in Neurobasal medium to a final concentration of 100 ng/ml, for the indicated period of time. When appropriate, 1.5 µM α-amanitin or actinomycin D (transcription inhibitors), 2.0 µM emetine or anisomycin (translation inhibitors) (Calbiochem), 200 nM K252a (TrkB inhibitor), 5 µM U73122 (PLCγ pathway inhibitor), 5 µM chelerythrine (PKC inhibitor) or 1 µM KN-93 (CAMKII inhibitor), 20 µM PD098059 or 10 µM U0126 (Ras-ERK pathway inhibitors), 30 µM LY294002 or 300 nM Wortmannin (PI3K/Akt pathway inhibitors) (Sigma-Aldrich Química) were added 30 min before BDNF stimulation, as indicated. The cells were further incubated with the signaling inhibitors for 3 h or 5 h, during BDNF stimulation. When appropriate, 100 ng/ml IGF-1 (insulin-like growth factor 1) and bFGF (basic fibroblast growth factor) (Sigma-Aldrich Química) were added in lieu of BDNF.

### Preparation of Extracts

Hippocampal neurons (DIV7/DIV14) were washed twice with ice-cold PBS and once more with PBS supplemented with 1 mM DTT and a cocktail of protease inhibitors (0.1 mM PMSF; CLAP: 1 µg/ml chymostatin, 1 µg/ml leupeptin, 1 µg/ml antipain, 1 µg/ml pepstatin; Sigma-Aldrich Química). The cells were then lysed with RIPA buffer (150 mM NaCl, 50 mM Tris-HCl, 5 mM EGTA, 1% Triton, 0.5% DOC and 0.1% SDS at a final pH 7.5), supplemented with 50 mM NaF, 1.5 mM sodium orthovanadate and a cocktail of protease inhibitors, and sonicated, on ice, using an ultrasonic cell disrupter microtip (VibraCell, Sonics & Materials, Inc.), with 2 cycles of 10 consecutive 1 s, low-intensity pulses interspaced by 30 s, in order to fully disrupt membrane structure. After centrifugation at 16,100 g for 10 min, protein in the supernatants was quantified using the bicinchoninic acid (BCA) assay (Thermo Scientific), and the samples were denaturated with 2x concentrated denaturating buffer (125 mM Tris, pH 6.8, 100 mM glycine, 4% SDS, 200 mM DTT, 40% glycerol, 3 mM sodium orthovanadate, and 0.01% bromophenol blue), without denaturation at 95°C for 5 min, which would otherwise cause loss of vesicular proteins to the insoluble fraction.

### Total RNA Isolation and Reverse Transcription for Real-time PCR

Total RNA from cultured hippocampal neurons was extracted with TRIzol reagent (Invitrogen), according to the manufacturer’s instructions. The full content of a 6-well cell cluster plate, with 870,000 cells/well (DIV7), was collected for each experimental condition. For first strand cDNA synthesis, 3 µg of total RNA was reverse-transcribed with avian myeloblastosis (AMV) reverse transcriptase (Roche Applied Science) using random primers p(dN)_6_ (3.2 µg), dNTPs (1 mM each), MgCl_2_ (25 mM), RNase inhibitor (50 units) and gelatin (0.01 µg/µl) in reaction buffer (10 mM Tris, 50 mM KCl, pH 8.3), in a total volume of 40 µl. The reaction was performed at 25°C for 10 min, followed by 60 min at 42°C, for primer annealing to the RNA template and cDNA synthesis, respectively. The reverse transcriptase was then denatured during 5 min at 99°C, and the sample was cooled to 4°C for 5 min and finally stored at -80°C until further use.

### Real-time PCR

Real-Time PCR analysis of gene expression was performed using the LightCycler System II (Roche Applied Science). The PCR reactions were performed using LightCycler FastStart DNA Master SYBR Green I (Roche et al., 1996) in 20 µl capillaries. The primers used for amplification of genes encoding VGLUT1 and VGLUT2 were, respectively, VGLUT1, forward: 5′ TGG AGT TCC GGC AGG AGG AGT T; VGLUT1, reverse: 5′ GTG TGT GTG GTG ACT GGG CGC; VGLUT2, forward: 5′ GAA GAA ACG GGG GAC ATC ACT GAG A; VGLUT2, reverse: 5′ GTC TTG CGC ACT TTC TTG CAC AAA T. The primers used for the amplification of endogenous control gene 18S ribosomal RNA were those included in the Applied Biosystems TaqMan Ribosomal RNA Control Reagents Kit. Each primer of a pair was added to the reaction mixture (10 µl) at a final concentration of 0.8 µM, with 3 mM MgCl_2_, in addition to the “Hot Start” LightCycler Fast Start DNA Master SYBR Green I mix (1x) and 2.0 µl of cDNA sample. Thermal cycling was initiated with activation of the FastStart TaqDNA polymerase by denaturation during 10 min at 95°C followed by 45 cycles of a 30 s melting step at 95°C, a 5 s annealing step at 60°C, and a 25 s elongation step at 72°C. All temperature transition rates used were at 20°C/s. After amplification for 45 cycles, at least 10 cycles beyond the beginning of the linear phase of amplification, samples were subjected to a melting curve analysis according to the manufacturer’s instructions in order to confirm the absence of unspecific amplification products and primer-dimers. Samples containing no template were included as negative controls in all experiments.

### mRNA Quantitative Analysis

The mRNA levels of the constitutively expressed reference gene encoding 18S ribosomal RNA were used as a control, in all experiments. The relative changes in the mRNA levels of glutamate receptor subunits in cultured hippocampal neurons were determined using the ΔΔ*C*_p_ method. Accordingly, for each experimental condition (unstimulated neurons and neurons treated with 100 ng/ml BDNF for 30 min or 3 h) the “crossing point” (*C*_p_) values given by the LightCycler system II software, for each target gene, were subtracted by the respective *C*_p_ value determined for the 18S gene from the same sample and condition (Δ*C*_p_). This allows normalizing changes in target gene expression. Afterward, the Δ*C*_p_ values were subtracted by the respective values of the control for the target gene giving ΔΔ*C*_p_. The derivation to the value of 2^−(ΔΔ*C*p)^ sets each control at the unity (or 100%), because ΔΔ*C*_p_ (control) = 0, and the stimuli conditions used were set at percentage relative to control.

### Immunoblotting

Protein samples were separated by SDS-PAGE, in 12% polyacrylamide gels, transferred to polyvinylidene (PVDF) membranes (Millipore Corp.), and immunoblotted. Blots were incubated with primary antibodies (overnight at 4°C), washed and exposed to alkaline phosphatase (ECF)-conjugated secondary antibody (1 h at room temperature). Alkaline phosphatase activity was visualized by enhanced chemifluorescence (ECF) on the Storm 860 Gel and Blot Imaging System (GE Healthcare). The following primary antibodies were used: anti-VGLUT1 and anti-VGLUT2 (1∶1000, Synaptic Systems); anti-β-Tubulin I (1∶10000, Sigma-Aldrich Química), anti-β-actin I (1∶20000, Sigma-Aldrich Química), anti-pERK1/2 (1∶1000, Cell Signaling), anti-BDNF (1∶1000, Santa Cruz Biotechnology). Anti-rabbit or anti-mouse IgG alkaline phosphatase-conjugated secondary antibodies (respectively, 1∶20000 and 1∶10000, GE Healthcare) were used for detection.

### Immunocytochemistry

For immunocytochemistry, cultured hippocampal neurons were grown on poly-D-lysine coated glass coverslips, at a density of 80000 cells/well (12-well plates), and were then fixed in PBS supplemented with 4% paraformaldehyde/4% sucrose, for 15 min at room temperature. After fixation the cells were washed and permeabilized with 0.25% Triton X-100 in PBS, for 5 min at 4°C, washed once in PBS for 5 min, and then blocked with 10% BSA, for 1 h at room temperature, and stained with specific primary antibodies overnight at 4°C. The following primary antibodies were used: rabbit anti-VGLUT1 or anti-VGLUT2 (1∶1000 and 1∶500, respectively; Synaptic Systems) and mouse anti-β-Tubulin I (1∶1000; Sigma-Aldrich Química). Subsequently, cells were washed six times and incubated for 1 h at 37°C with the secondary antibodies (Alexa Fluor® 488 goat anti-rabbit and Alexa Fluor® 568 goat anti-mouse, 1∶500; Invitrogen). The cells were washed six times, mounted on glass slides with the Dako mounting medium and viewed on an Axio Observer 2.1 fluorescence microscope coupled to an Axiocam HRm digital camera. For each set of experiments the cell images were acquired using identical exposure settings. The regions of interest for the quantification were blindly chosen using the tubulin channel. The images were analyzed for the number, the integrated density (mean intensity×puncta area), and the intensity of VGLUT puncta along neurites, as well as the total immunoreactivity in the soma, using the ImageJ software (NIH). The quantification was performed after determination of the threshold and subtraction of the background. The results of the quantification were normalized for the length of the region of interest in the case of neurites or for the area in the case of the soma. At least 12 cells per condition were analyzed for each preparation.

### Statistical Analysis

Data are presented as mean ± SEM of at least three different experiments, performed in independent preparations. Statistical analysis was performed using one-way analysis of variance (ANOVA) followed by the Dunnett’s or Bonferroni post-tests, at a 99% confidence interval, or using the Student’s *t* test, as indicated in the figure captions.

## Results

### BDNF Upregulates VGLUT1 and VGLUT2 Total Protein Levels

**Figure 1:**
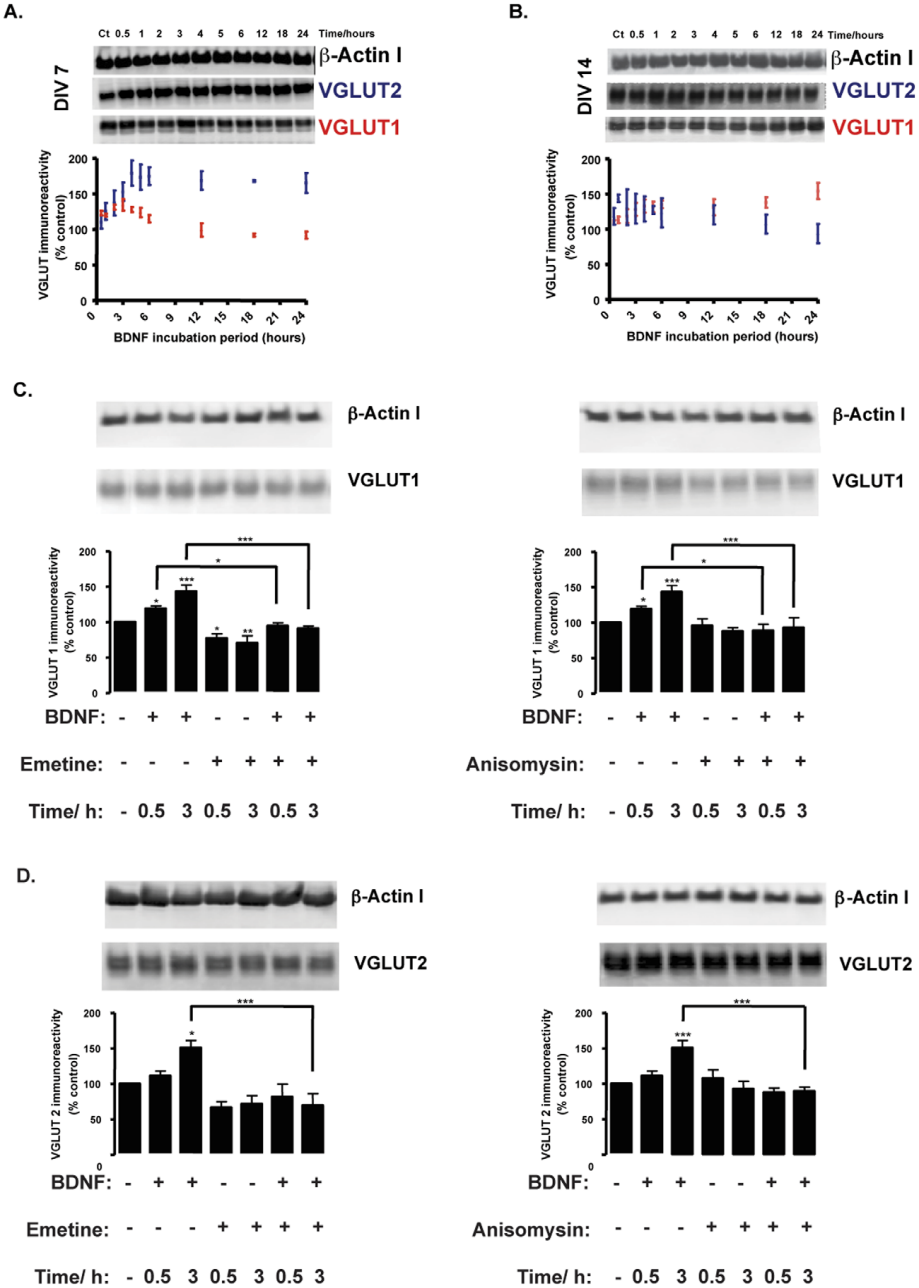
BDNF upregulates VGLUT1 and VGLUT2 protein expression through a translation-dependent mechanism. (A–B) Cultured hippocampal neurons at DIV7 (A) and DIV14 (B) were incubated with 100 ng/ml BDNF for different time periods and total VLGUT1 and VGLUT2 protein levels were compared to control (without BDNF) expression, upon normalization with β-actin I levels. (C–D) Cultured hippocampal neurons at DIV7 were pre-incubated or not with the translation inhibitors emetine or anisomycin (2 µM) for 30 min before BDNF stimulation during 30 min or 3 h and VGLUT1 (C) and VGLUT2 (D) protein levels were compared to control expression. When the effect of translation inhibitors was tested, the cells were incubated with the compounds during stimulation with BDNF. (A–D) Quantification of 3–5 different experiments, performed in independent preparations, is presented as mean percentage ± SEM compared to the control (unstimulated neurons). Statistical significance was determined by One Way ANOVA followed by Bonferronís multiple comparison test with a confidence interval of 99% (*p<0.05, **p<0.01, ***p<0.001).

7 and 14 DIV cultured hippocampal neurons were incubated with or without 100 ng/ml BDNF, for different time periods (30 min to 24 h), in order to determine whether acute stimulation with BDNF affects the protein expression of vesicular glutamate transporters. VGLUT1 and VGLUT2 protein levels were determined by Western blotting (Fig. 1A). At DIV7, BDNF rapidly and significantly upregulated VGLUT2 protein levels, while VGLUT1 protein levels were only transiently upregulated at the initial time points, subsequently returning to levels similar to the control condition (unstimulated neurons). In contrast, at DIV14, BDNF did not significantly change VGLUT2 protein levels but instead upregulated VGLUT1 throughout time (Fig. 1B). The increase in VGLUT2 protein levels at DIV7 and in VGLUT1 at DIV14 had distinct kinetics, and the maximal effects were found after incubation with BDNF for 3 h and 24 h, respectively. At DIV7, after a rapid increase, VGLUT2 protein levels remained high and relatively similar to the maximal value (3 h), even 24 h after incubation, while the abundance of VGLUT1 showed a slow and gradual increase, in comparison to the control. The sustained increase in VGLUT1 and VGLUT2 protein levels observed in hippocampal neurons (DIV14 and DIV7, respectively) subjected to a chronic stimulation with BDNF was not observed when the incubation was limited to 4 h, and followed by 14 h incubation in culture conditioned medium (p>0.05) (Fig. S1). The effect of BDNF on VGLUT2 expression was not further examined at DIV14 because it was not significant and the endogenous expression levels of this isoform are rather low and variable, in developed neurons. These results mimic the developmental switch, from VGLUT2 to VGLUT1 expression, observed in postnatal hippocampal neurons [42], [43], [48].

In order to test whether the effect of BDNF resulted from an increase in protein synthesis, we used two translation inhibitors, anisomycin and emetine. Hippocampal neurons were stimulated with BDNF for 30 min or 3 h, in the presence or absence of translation inhibitors, which were added to the cultured media 30 min prior to BDNF stimulation and kept in the media during incubation with BDNF. Emetine (2 µM) or anisomycin (2 µM) fully abrogated the effect of BDNF on VGLUT1 (Fig. 1C) and VGLUT2 (Fig. 1D) isoforms at DIV7. None of the protein synthesis inhibitors reduced VGLUT1 or VGLUT2 protein levels under control conditions (p>0.05), in agreement with the relatively long half-life suggested for VGLUT2 [49]. However, translation inhibition was not tested at DIV14 because BDNF only upregulates VGLUT1 protein levels for long incubation periods, above the cellular toxicity threshold of emetine and anisomycin [50]. Treatment with anisomycin or emetine alone did not alter VGLUT1 or VGLUT2 protein levels in the time periods tested (p>0.05). Taken together, these results indicate that BDNF upregulates VGLUT isoforms 1 and 2 through a protein-synthesis dependent mechanism, and rule out the hypothesis of a reduction in protein degradation.

### BDNF Upregulates VGLUT1 and VGLUT2 by Enhancing Transcriptional Activity

**Figure 2:**
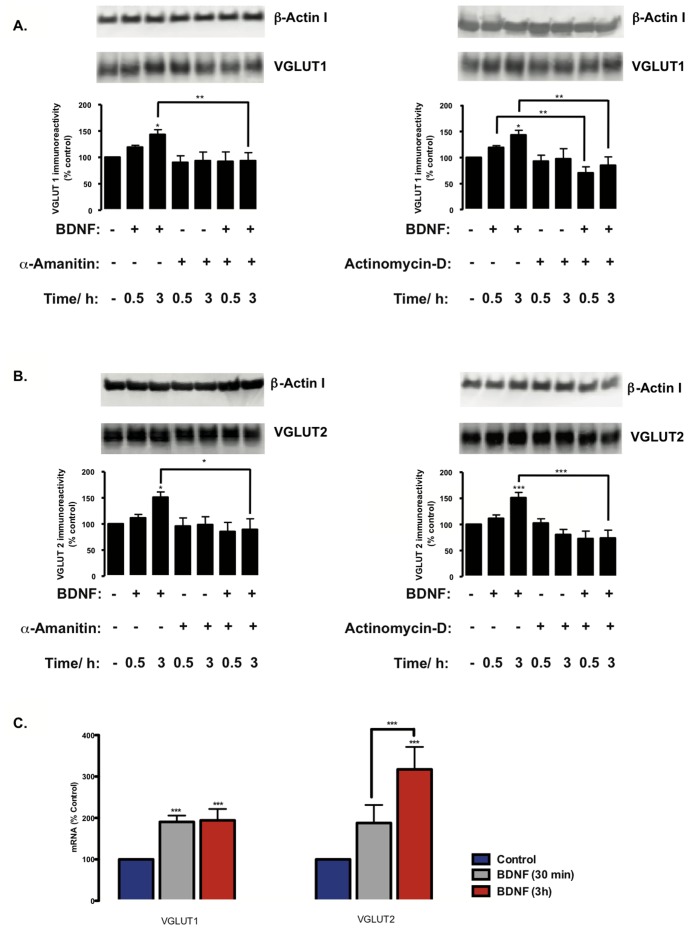
The effect of BDNF on VGLUT1 and VGLUT2 protein levels is dependent on gene expression. (A–B) Hippocampal neurons were stimulated with BDNF (100 ng/ml) for the indicated periods of time, in the presence or in the absence of the transcription inhibitors α-amanitin (1.5 µM) or actinomycin-D (1.5 µM), and VGLUT1 (A) and VGLUT2 (B) protein levels were determined by western blot. (C) The variation of *Slc17a7* (VGLUT1) and *Slc17a6* (VGLUT2) mRNA levels was assayed by real-time PCR, as described in the methods section. The neurons were stimulated with 100 ng/ml BDNF during 30 minutes (grey) or 3 hours (red). (A–C) Quantification of 4–5 experiments, performed in independent preparations, is presented as mean percentage ± SEM compared to the control (unstimulated neurons), and normalized to *18S* reference gene. Statistical significance was determined by One Way ANOVA followed by Bonferronís multiple comparison test with a confidence interval of 99% (*p<0.05, **p<0.01, ***p<0.001).

BDNF signaling may stimulate gene transcription [51] and/or protein synthesis [52], [53]. Hence, we used two different transcription inhibitors, α-amanitin (1.5 µM) and actinomycin D (1.5 µM) to test the role of transcription in the upregulation of vesicular glutamate transporters by BDNF. Both transcription inhibitors blocked the effect of BDNF on VGLUT1 (Fig. 2A) and VGLUT2 (Fig. 2B) protein levels and had no effect on the abundance of VGLUT variants in the absence of this neurotrophin, relative to the control condition. In agreement with these findings, real-time PCR experiments showed that BDNF stimulation for 30 min caused an approximately 2-fold increased in VGLUT1 mRNA levels (p<0.001). A significant increase in VGLUT2 mRNA (approximately 3-fold) was also found when cells were incubated with the neurotrophin for 3 h (p<0.001), with VGLUT1 mRNA levels remaining at a similar level to that of stimulation for only 30 min (Fig. 2C). Overall, the results suggest that BDNF regulates VGLUT1 and VGLUT2 gene expression, likely through activation of a BDNF signaling-modulated transcription mechanism and/or transcription factor(s).

### VGLUT Upregulation Depends on TrkB Receptor Activation Specifically Induced by BDNF

**Figure 3:**
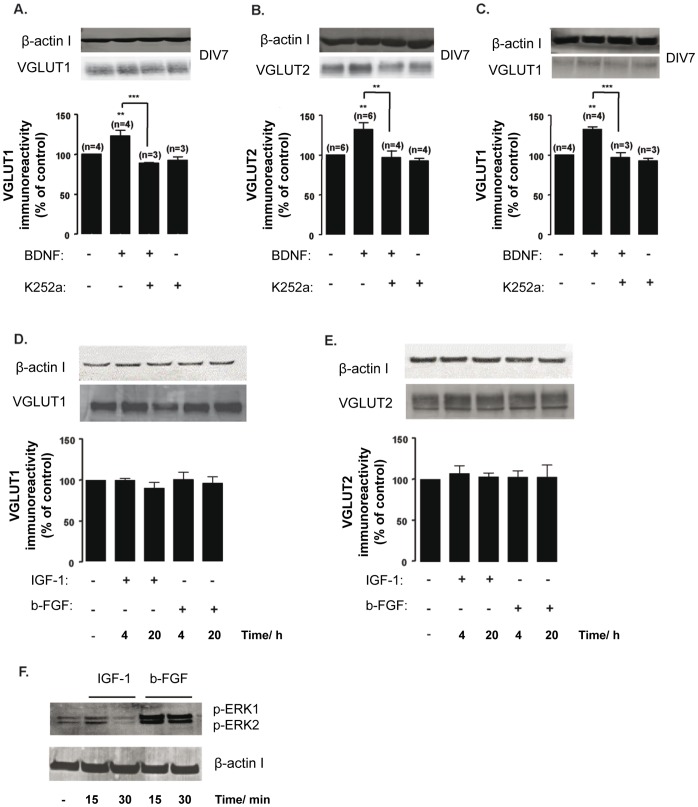
TrkB receptor inhibition blocks BDNF upregulation of VGLUT1 and VGLUT2. (A–C) Cultured hippocampal neurons at DIV7 (A, B) and DIV14 (C) were stimulated with BDNF (100 ng/ml), for the indicated periods of time, in the presence or absence of a selective inhibitor of tyrosine kinase activity, K252a (200 nM), and VGLUT1 (A, C) and VGLUT2 (B) protein levels were determined by western blot. Quantification of the indicated number of experiments, performed in independent preparations, is presented as mean percentage ± SEM compared to the control (unstimulated neurons). (D–F) DIV7 hippocampal neurons were stimulated with IGF-1 or bFGF, for 4 or 20 h, and VGLUT1 (D) and VGLUT2 (E) protein levels were determined by western blot. Quantification of 4 different experiments, performed in independent preparations, is presented as mean percentage ± SEM compared to the control. Statistical significance was determined by One Way ANOVA followed by Bonferronís multiple comparison test with a confidence interval of 99% (*p<0.05, **p<0.01, ***p<0.001). (F) DIV7 hippocampal neurons were stimulated with IGF-1 or bFGF, for 15 or 30 min, and the levels of ERK1/2 phosphorylation were determined by western blot. The antibody used specifically recognizes the phosphorylated isoforms 1 and 2 of ERK, but not the nonphosphorylated (presumably inactive) proteins.

BDNF signaling may stimulate gene transcription [51] and/or protein synthesis [52], [53], essentially through activation of TrkB receptors [33]. K252a is a potent inhibitor of tyrosine protein kinase activity of TrkA, TrkB and TrkC receptors, blocking receptor autophosphorylation and, consequently, the biological functions of their neurotrophin ligands [54]. In addition to TrkB, cultures of embryonic day 18 (E18) hippocampal neurons express TrkC, but not TrkA receptors [55] and BDNF does not bind to TrkC receptors [56], [57]. The results found show that TrkB receptor activation is required for upregulation of VGLUT1 (DIV7 and DIV14) and VGLUT2 (DIV7) protein levels because no effect of BDNF was found when the stimulation with the neurotrophin was performed in the presence of 200 nM K252a (p>0.05) (Fig. 3A–C). K252a alone did not significantly alter either VGLUT1 or VGLUT2 protein levels, when compared to the control condition (without treatment) (p>0.05), which demonstrates a specific action of BDNF in the upregulation of VGLUT expression (Fig. 3A–C). In addition, the lack of effect of K252a on VGLUT2 expression at DIV14 did not result from the endogenous release of saturating amounts of BDNF, which would prevent any additional effect by its exogenous application, because incubation with the Trk receptor inhibitor alone did not decrease VGLUT2 protein expression below the control levels (Fig. S2). This further suggests that BDNF signaling may regulate the developmental switch from VGLUT2 to VGLUT1 expression in hippocampal neurons.

Cultured hippocampal neurons express receptors for other trophic factors, including IGF-1 [58], [59] and bFGF [60], [61], which activate the same BDNF-induced signaling pathways in cultured hippocampal neurons. Moreover, IGF-I enhances the expression of TrkB receptors and the ability of BDNF to induce ERK1/2 phosphorylation in cerebrocortical neurons [62] while bFGF rapidly stimulates BDNF expression in the hippocampal cell line HiB5 [63]. In this context, we tested whether acute stimulation with IGF-1 or bFGF in lieu of BDNF would affect the expression levels of VGLUT isoforms, at two different time points (4 h and 20 h). Either brief or prolonged incubation with 100 ng/ml IGF-1 or bFGF had no effect on VGLUT1 (Fig. 3D) and VGLUT2 (Fig. 3E) protein levels, at DIV7, when compared to the control condition (p>0.05). Since exogenously applied neurotrophic factors are only effective when their receptors are expressed at the cell surface and free to bind their ligands, the absence of any effect on VGLUT expression could have resulted from ligand or receptor inactivity. In order to exclude this possibility, we tested the levels of ERK1/2 phosphorylation, upon 15 or 30 min of stimulation with IGF-1 or bFGF. The antibody used specifically recognizes the phosphorylated isoforms 1 and 2 of ERK, but not the non-phosphorylated (presumably inactive) proteins. The results show ERK1/2 phosphorylation after 15 min incubation with both IGF-1 and bFGF, and the effect of bFGF was still observed after 30 min of stimulation. These results confirm that the lack of effect of IGF-1 and bFGF on VGLUT expression was not due to inactivity of trophic factors or their receptors (Fig. 3F). Therefore, we may conclude that BDNF upregulates VGLUT1 and VGLUT2 specifically through activation of TrkB receptors as K252a fully abrogated the effect in cultured hippocampal neurons at DIV7 (Fig. 3A, B) and DIV14 for VGLUT1 (Fig. 3C).

### BDNF Regulates VGLUT Expression through PLCγ Signaling Pathway Activation

**Figure 4:**
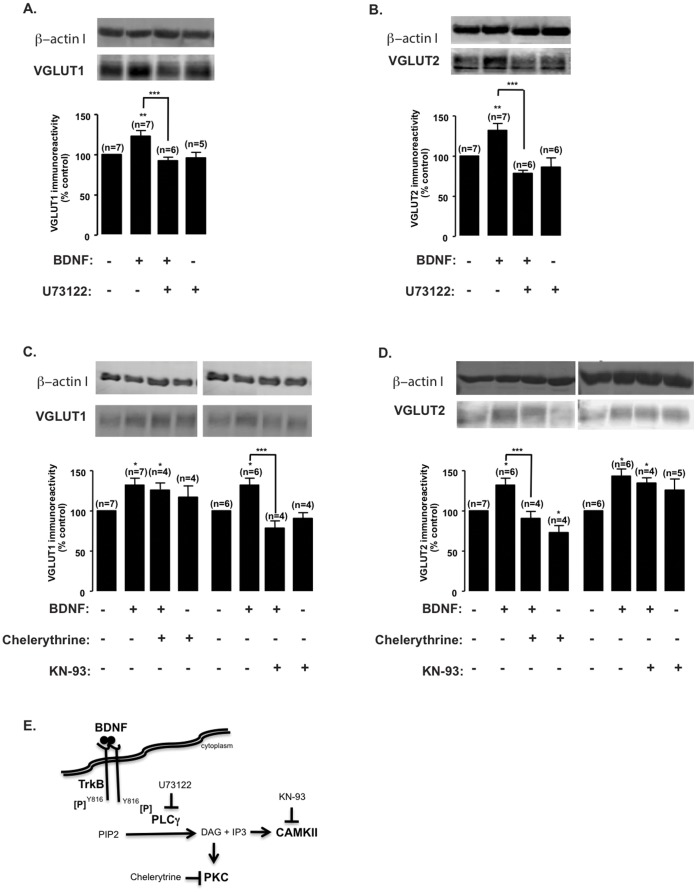
Inhibition of the PLCγ signaling pathway blocks BDNF-induced upregulation of VGLUT1 and VGLUT2 protein levels. (A–D) DIV7 cultured hippocampal neurons were stimulated with BDNF (100 ng/ml) for the indicated periods of time, in the presence or absence of U73122 (PLCγ inhibitor; 5 µM) (A, B), chelerytrine (PKC inhibitor; 5 µM) or KN-93 (CAMKII inhibitor; 1 µM) (C, D), and VGLUT1 (A, C) and VGLUT2 (B, D) protein levels were determined by western blot. Quantification of the indicated number of experiments, performed in independent preparations, is presented as mean percentage ± SEM compared to the control (unstimulated neurons). Statistical significance was determined by One Way ANOVA followed by Bonferronís multiple comparison test with a confidence interval of 99% (*p<0.05, **p<0.01, ***p<0.001). (E) Schematic representation of BDNF-induced TrkB receptor trans-activation and downstream PLCγ signaling pathway effectors and inhibitors.

**Figure 5:**
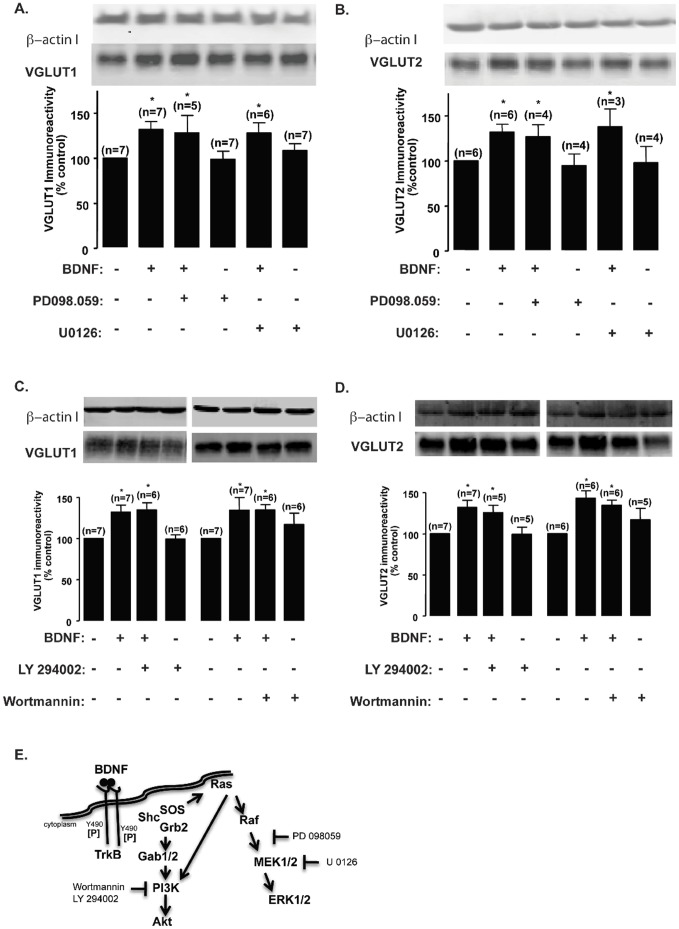
Inhibition of the PI3-K/Akt and Ras-ERK signaling pathways has no significant effect on BDNF-induced upregulation of VGLUT1 and VGLUT2 protein levels. (A–D) DIV7 cultured hippocampal neurons were stimulated with BDNF (100 ng/ml), for the indicated periods of time, in the presence or absence of Ras-ERK pathway inhibitors PD098059 (20 µM) or U0126 (10 µM) (A, B), or PI3K/Akt inhibitors LY294002 (30 µM) or Wortmannin (300 nM) (C, D), and VGLUT1 (A, C) and VGLUT2 (B, D) protein levels were determined by western blot. Quantification of the indicated number of experiments, performed in independent preparations, is presented as mean percentage ± SEM compared to the control (unstimulated neurons). Statistical significance was determined by One Way ANOVA followed by Bonferronís multiple comparison test with a confidence interval of 99% (*p<0.05, **p<0.01, ***p<0.001). (E) Schematic representation of BDNF-induced TrkB receptor trans-activation and downstream effectors and inhibitors of the PI3-K/Akt and Ras-ERK signaling pathways.

The specificity of BDNF signaling through TrkB activation prompted us to further assess which pathway(s) triggered by TrkB trans-autophosphorylation was (were) involved in BDNF-mediated VGLUT upregulation. For this purpose, we used the chemical inhibitors U73122 (PLCγ pathway), PD098059 or U0126 (Ras-ERK pathway), and LY294002 or Wortmannin (PI3K/Akt pathway). At DIV7, U73122 fully abrogated BDNF-induced VGLUT1 (Fig. 4A) and VGLUT2 (Fig. 4B) upregulation (p>0.05), indicating that this pathway plays a key role in response to BDNF. Chemical inhibitors chelerythrine and KN-93 selectively and potently block the activation of PKC and CAMKII, respectively, two kinases that act downstream of PLCγ. We have found that incubation with KN-93 (1 µM) prevented BDNF-induced VGLUT1 (p>0.05), but not VGLUT2 upregulation (p<0.05), while chelerythrin (5 µM) blocked VGLUT2 upregulation (p>0.05), but was without effect on VGLUT1 (p<0.05) (Fig. 4C, D). These results indicate that BDNF regulates VGLUT 1 and 2 expression through signaling mechanisms acting downstream of PLCγ. VGLUT1 transient upregulation at DIV7 is dependent on CAMKII activation whereas VGLUT2 long lasting upregulation, at the same developmental stage, requires PKC activation. Blocking the Ras-ERK (Fig. 5A, B) or PI3-K/Akt (Fig. 5C, D) signaling pathways with PD098059 or U0126 and LY294002 or Wortmannin, respectively, showed no significant effect (p>0.05).

### BDNF Affects VGLUT Subcellular Distribution

**Figure 6:**
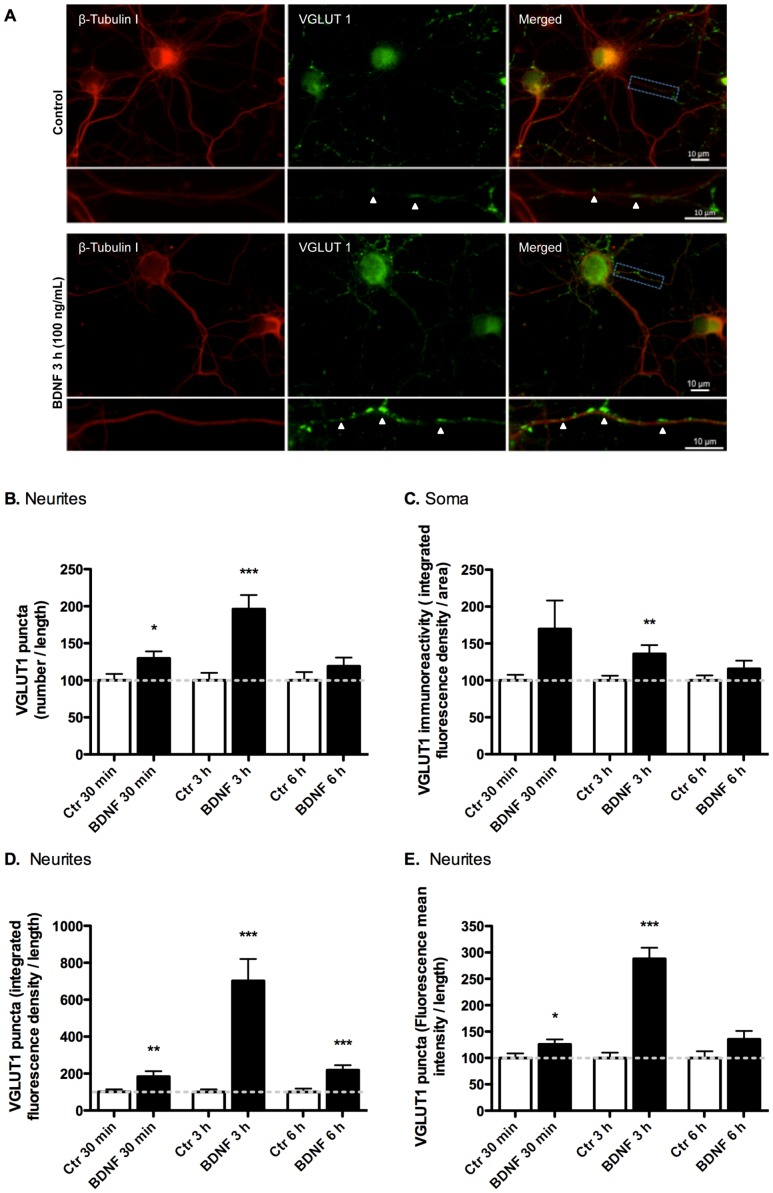
Effect of BDNF on the subcellular distribution of VGLUT1 in cultured rat hippocampal neurons. Hippocampal neurons were stimulated at 7 DIV with BDNF (100 ng/ml) for 30 min, 3 h or 6 h. Neurons were then stained for total VGLUT1 (green) and β-tubulin I (red) (A) (Scale bar: 10 µm). Arrowheads show the location of VGLUT1 puncta. The acquired fluorescence images were analysed to assess the number (B), integrated density (mean intensity×puncta area) (D) and intensity (E) of VGLUT1 puncta in neurites, as well as for VGLUT1 immunoreactivity in the soma (C). Results were normalized for neuritic length (B, D and E) or for soma area (C). The protein localization was visualized using a Zeiss Axio Observer 2.1 fluorescence microscope (63x Objective). Quantitative particle analysis was performed using ImageJ software. Results are shown as mean percentage of control of at least three independent experiments (n ≥30 cells per condition). *p<0.05; **p<0.01; ***p<0.001, significantly different in comparison to the respective control (unpaired Student’s *t*-test).

**Figure 7:**
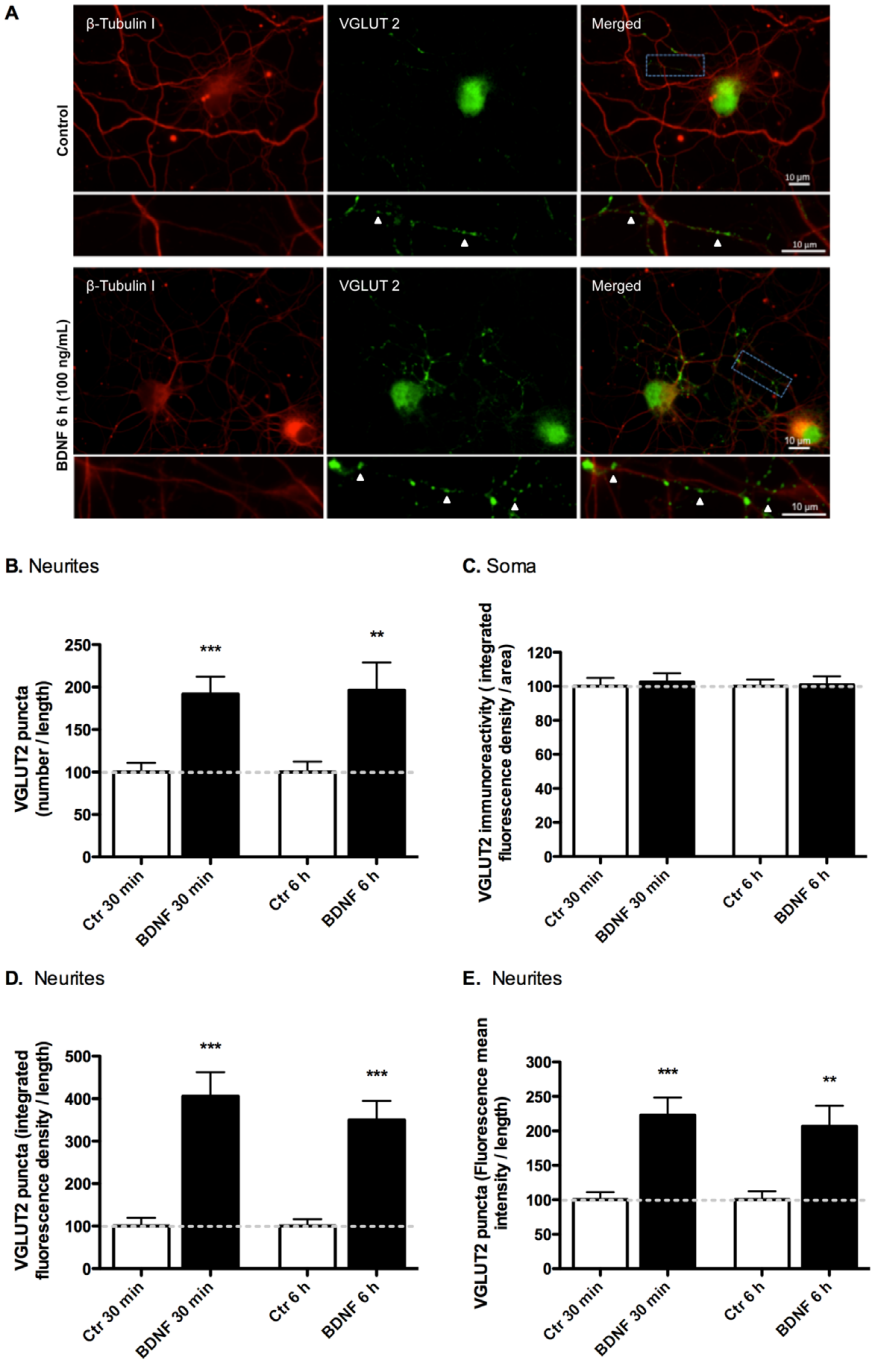
Effect of BDNF on the subcellular distribution of VGLUT2 in cultured rat hippocampal neurons. Hippocampal neurons at 7 DIV were stimulated with BDNF (100 ng/ml) for 30 min or 6 h. Neurons were immunolabeled with specific antibodies for total VGLUT2 (green) and β-tubulin I (red) (A) (Scale bar: 10 µm). Arrowheads show the location of VGLUT2 puncta. The acquired fluorescence images were analysed to assess the number (B), integrated density (mean intensity×puncta area) (D) and intensity (E) of VGLUT2 puncta in neurites, as well as for VGLUT1 immunoreactivity in the soma (C). Results were normalized for neuritic length (B, D and E) or soma area (C). The protein localization was visualized using a Zeiss Axio Observer 2.1 fluorescence microscope (63x Objective). Quantitative particle analysis was performed using ImageJ software. Results are shown as mean percentage of control of at least three independent experiments (n ≥30 cells per condition). **p<0.01; ***p<0.001, significantly different in comparison to the respective control (unpaired Student’s *t*-test).

The subcellular distribution of VGLUT1 and VGLUT2 was assessed by immunocytochemistry in cultured hippocampal neurons (DIV7) stimulated or not with 100 ng/ml BDNF, for different time periods, and imaged by fluorescence microscopy. VGLUT1-positive neuritic (presumably axonal) labeling transiently increased after 30 min - 3 h of incubation with BDNF when compared with the control (Fig. 6A). Quantification of the immunofluorescence images showed a BDNF-induced increase in the number (Fig. 6B), integrated density (mean intensity×puncta area) (Fig. 6D) and intensity (Fig. 6E) of VGLUT1 puncta along neurites, as well as an upregulation in the total immunoreactivity in the soma (Fig. 6C). A small but non-significant upregulation of VGLUT1 protein levels was found in the soma, at an early time point (30 min), before the maximal increase in VGLUT1 puncta intensity (3 h) (p<0.001). VGLUT2 punctate labeling was also increased in the neurites of hippocampal neurons following stimulation with BDNF for 30 min or 6 h (Fig. 7). In this case, BDNF was also found to increase the number (Fig. 7B), integrated density (Fig. 7D) and intensity (Fig. 7E) of VGLUT2 puncta in neurites, with maximal effects at 30 min of incubation with the neurotrophin. However, BDNF was without effect on total VGLUT2 immunoreactivity in the soma (p>0.05) (Fig. 7C), in contrast with the results obtained for VGLUT1. Overall, these imaging results not only provide further support to the biochemistry results previously presented but also show that BDNF affects the subcellular distribution and trafficking of VGLUT1 and VGLUT2 in hippocampal neurons.

## Discussion

We and others have previously shown direct presynaptic effects of BDNF, which upregulates K^+^-evoked [^3^H] glutamate release from hippocampal synaptosomes, in a subset of glutamatergic synapses expressing TrkB receptors on the plasma membrane [39], [64]. In the current study, we have shown that BDNF regulates VGLUT1 and VGLUT2 gene and protein expression, during development of cultured hippocampal neurons, specifically through activation of TrkB receptors and the PLCγ signaling pathway. At DIV7, BDNF-induced transient VGLUT1 upregulation requires the activation of the PLCγ downstream effector CAMKII, whereas VGLUT2 sustained upregulation, at the same developmental stage, depends on PKC activation. At DIV14, BDNF upregulates VGLUT1 expression with no significant effect on VGLUT2 expression, which was endogenously downregulated during this period, approximately corresponding to the developmental switch from VGLUT2 to VGLUT1 neurons in postnatal hippocampus [42], [43]. The results also indicate that BDNF affects VGLUT1 and VGLUT2 subcellular distribution, further suggesting a role in BDNF-induced short-term plasticity and LTP. These findings correlate with the BDNF-induced increase in the number of docked vesicles at the active zone and quantal glutamate release observed at hippocampal excitatory synapses [39], [41], [65].

### Effect of BDNF on VGLUT1 and VGLUT2 Gene and Protein Expression

In this study, we show that BDNF differentially upregulates VGLUT isoforms 1 and 2 during the development of hippocampal neurons in a time-dependent manner (Fig. 1A), by a mechanism sensitive to inhibition of transcription (Fig. 2A–D) and translation (Fig. 1C, D). The BDNF-induced sustained increase in VGLUT2 protein levels contrasts with the transient upregulation of VGLUT1 protein levels induced by the neurotrophin. The latter effects correlate with the transient increase in TrkB signaling activity observed in hippocampal neurons incubated with BDNF, which reached a maximum after about 10 min of exposure to the neurotrophin and decreased to control levels after 24 h of incubation [3]. These results also indicate that VGLUT1 synthesized in response to BDNF stimulation is degraded within less than 24 h. The more sustained BDNF-induced increase in VGLUT2 protein levels suggests that this transporter has a longer half-life in hippocampal neurons than VGLUT1. Alternatively, the results may indicate that the TrkB signaling pathway coupled to the regulation of VGLUT2 expression (which is distinct from the pathway responsible for VGLUT1 upregulation) may undergo a slower inactivation after desensitization of the TrkB receptors. In addition to the effect on VGLUT protein levels reported here, BDNF was previously shown to upregulate the expression of the synaptic vesicle proteins synaptophysin, synaptobrevin and synaptotagmin, but showed no effect on the presynaptic membrane proteins syntaxin and SNAP-25, or the vesicle-binding protein synapsin-I, in organotypical cultures of hippocampal neurons [66]. BDNF overexpression in a Huntington’s disease mouse model was previously shown to prevent the decrease of striatal VGLUT1, but that effect most likely resulted from a neuroprotective mechanism of BDNF, which may have precluded the loss of glutamatergic synapses [67].

Immunocytochemistry experiments also showed a rapid effect of BDNF on VGLUT1 protein levels in the soma, which was followed by an increase in the expression of this protein in puncta along neurites (presumably axons). These results suggest that newly synthesized vesicular glutamate transporters are delivered to neurites within 3 h, being clustered in both new and pre-existing puncta. BDNF also induced a sustained increase in the intensity of VGLUT2 puncta in neurites, but no changes were found in the somatic abundance of the transporter. This suggests that VGLUT2 synthesized in the soma following BDNF stimulation may be rapidly delivered to neurites or, alternatively, VGLUT2 may be produced locally at the neurites in response to stimulation with the neurotrophin. Furthermore, the increase noted in the number of VGLUT1/2 puncta and in the number of transporters clustered in these regions, in BDNF-stimulated hippocampal neurons may result, at least partly, from a redistribution of vesicles containing the vesicular transporters already available in neurites.

VGLUT1 and VGLUT2 were initially identified as Na^+^-dependent inorganic phosphate transporters BNPI and DNPI [68]–[70] in screenings of cDNAs upregulated by NMDA and growth factors, respectively [53]. However, the characterization of VGLUT1 [71], [72] and VGLUT2 promoters [73] has only recently been performed, and no transcription factors or signaling pathways directly modulating the expression of these genes have been identified thus far, to our knowledge. Nonetheless, VGLUT1 protein levels show strong diurnal cycling, which is lost in mice lacking the period gene *Period 2* [74]. CAMKII is maximally active during the subjective day, in contrast to Erk [75], and the CAMKII inhibitor KN-93 was shown to block *Period 2* expression while the MEK inhibitors PD98059 and U0126 were without effect [76]. In agreement with these results, we have found that CAMKII inhibition blocks BDNF-induced upregulation of VGLUT1 (Fig. 4C), whereas MEK/ERK inhibition had no significant effect (Fig. 5A). VGLUT1 is also upregulated in cerebrocortical and hippocampal regions of rat brains upon antidepressant treatment with fluoxetine, paroxetine or desipramine [77], or in striatal neurons due to intraperitoneal injection of methanphetamine [78]. In turn, VGLUT2 is upregulated in vasopressin and oxytocin neurons after osmotic stimulation [79] or the thalamus of schizophrenic patients [80] although, in all cases, the underlying transcriptional mechanisms are still unknown. Nevertheless, these results demonstrate that glutamatergic neurons regulate glutamate release through modulation of VGLUT expression, which is endogenously regulated in developing and mature neurons enabling synaptic refinement and plasticity [44].

### TrkB Activation and PLCγ Signaling in BDNF-mediated Regulation of Glutamatergic Function

We have found that BDNF regulation of VGLUT expression in developing hippocampal neurons depends specifically on the activation of TrkB receptors and PLCγ signaling (Fig. 4), and although stimulation with BDNF is also coupled to the activation of the Ras/ERK and PI3-K signaling pathways in cultured hippocampal neurons [3] these pathways do not participate in the regulation of VGLUT1 and VGLUT2 expression. Interestingly, the effects of BDNF are specific since stimulation of cultured hippocampal neurons with IGF-1 and bFGF, which also activate receptor tyrosine kinases, did not affect VGLUT protein levels. This difference may be due to a distinct location of the receptors in the cells and/or to a differential coupling to intracellular signaling mechanisms. In cultured cerebrocortical neurons, TrkB receptors were found in all major compartments of each neuron (cell bodies, dendrites, and axons) both before (DIV4) and during the peak of (DIV10) synapse formation [81].

The role of PLCγ signaling in BDNF-induced upregulation of VGLUT protein levels in cultured hippocampal neurons correlates with its role in the modulation of other components of glutamatergic synapses by BDNF. BDNF-induced glutamate release depends on the PLCγ pathway [82], [83] and ceases following treatment with a synthetic glucocorticoid (DEX) that decreases glucocorticoid receptor-TrkB interaction thereby attenuating PLCγ activation [84]. Likewise, in cultured hippocampal neurons, BDNF enhances glutamatergic synaptic transmission by raising the presynaptic intracellular calcium concentration, due to Ca^2+^ release from IP3-sensitive stores [85]. Furthermore, in hippocampal synaptosomes, the effect of BDNF on K^+^-evoked [^3^H] glutamate release correlates with increased PLCγ phosphorylation but not ERK or Akt phosphorylation [64]. Additionally, both the early and late phases of long-term potentiation are impaired in the CA1 hippocampus region of homozygous mice with mutant PLCγ docking sites at TrkB receptors, as a result of impaired CAMKIV and CREB phosphorylation, whilst mutation of Shc docking site, upstream of Ras-Erk and PI3K/Akt, had no effect on LTP [18]. The effects of BDNF on LTP are likely mediated by activation of pre- and post-synaptic TrkB receptors since selectively blocking of pre- or postsynaptic signaling showed no significant reduction in LTP [22], [23]. The current results showing BDNF-induced differential upregulation of VGLUT1 and VGLUT2, via CAMKII and PKC activation, respectively, identify VGLUT as potential presynaptic molecular targets of BDNF contribution to protein-synthesis dependent mechanisms of synaptic plasticity. This is supported by evidences showing that inhibition of BDNF signaling impairs LTM [86], [87], VGLUT1 deletion results in impaired LTP, learning and memory function [46], [47], and both isoforms are crucial effectors of synaptic plasticity [42], [88].

In addition to the effects on VGLUT protein levels, activation of TrkB receptors by BDNF has also been shown to enhance glutamate release in cultured hippocampal neurons by increasing the frequency of miniature excitatory postsynaptic currents (mEPSCs) [85], [89]. Other authors have also reported that BDNF enhances presynaptic function by increasing the number of docked vesicles at the active zone and quantal glutamate release [39], [41] when the postsynaptic neuron is glutamatergic or excitatory but not when GABAergic or inhibitory [90]. At the postsynaptic level, BDNF may potentiate excitatory synaptic transmission by regulating the expression and synaptic delivery of AMPA receptor subunit GluA1, through activation of PKC and CAMKII [37], and upregulating the expression of GluN1, GluN2A and GluN2B NMDA subunits in a transcription-dependent mechanism [38]. CAMKII and PKC, activated downstream of BDNF binding to TrkB receptors and PLCγ stimulation, have a key role in the potentiation of NMDA receptors by BDNF [91], [92]. These findings support the model whereby BDNF induces LTP through targeting of both pre- and postsynaptic mechanisms, critical for synaptic function. We have also found that incubation with BDNF has no effect on protein markers of GABAergic neurons, glutamate decarboxylase 65 and 67 (data not shown), further demonstrating the correlation between BDNF signaling-dependent regulation of neuronal protein levels and function.

In addition to TrkB receptors, BDNF may also bind to p75^NTR^, abundantly expressed in the hippocampus during the late embryonic and early postnatal [93], [94] period of developmental cell death [95], [96], although with low affinity [19]. Furthermore, BDNF binds to truncated TrkB receptors, but their endogenous expression does not peak until postnatal days 10–15 (P10–15), in contrast with the full-length (FL) TrkB mRNA, which reaches adult levels at birth (P0) [97]. Hence, BDNF signaling in developing hippocampal neurons is essentially dependent on TrkB.FL receptor activation. This is in accordance with the results reported here showing that BDNF-induced upregulation of VGLUT was inhibited by the Trk receptor inhibitor K252a.

### VGLUT1 and VGLUT2 may Mediate BDNF-induced Mechanisms of Synaptic Plasticity

The current study showing a BDNF-induced upregulation of total VGLUT protein levels in hippocampal neurons, in addition to an increase in the punctate distribution of the transporters along neurites, provides further evidence indicating a role of this neurotrophin on presynaptic potentiation of glutamatergic transmission. The following evidences suggest that BDNF-induced upregulation in VGLUT clustering in neurites may significantly potentiate excitatory neurotransmission: 1) VGLUT expression directly correlates with synaptic strength [43], [44] and biogenesis or recycling of synaptic vesicles [42], [98]; 2) VGLUT1 deficient mice exhibit decreased spontaneous glutamate release and quantal synaptic transmission due to exocytosis of partially filled vesicles in hippocampal synapses [43]; 3) VGLUT1 overexpression not only rescues this phenotype but also enhances AMPA receptor-mediated evoked EPSCs by increasing glutamate release per vesicle [44]; 4) loss of VGLUT 1 and 2 causes changes in synaptic vesicle shape and leads to decreased number of vesicles [42], [98]; 5) VGLUT2 deficiency decreases evoked glutamate release probability and reduces LTD at hippocampal CA3-CA1 synapses of young postnatal (P11–P14) mice [99]; 6) even though one transporter apparently suffices to fill a vesicle [100], enhanced VGLUT expression may increase the number of transporters per vesicle, thus, accelerating the rate of vesicle filling or its volume [101]. Conversely, decreased VGLUT1 expression causes depressive behavior and impaired memory in mice [47], while VGLUT2 heterozygotes show decreased neuropathic pain and defense responses [98], [102]. Hence, differences between transcription and translation rates or synaptic delivery of VGLUT isoforms, otherwise quite similar in function, further explain presynaptic regulation of quantal size.

In conclusion, the results presented herein suggest that BDNF signaling regulates differentially the gene and protein expression of VGLUT1 and VGLUT2 in developing and mature hippocampal neurons. Nevertheless, future in vivo studies as required for verifying the potential role of BDNF-mediated regulation of VGLUT expression in hippocampal synaptic mechanisms of short-term plasticity and long-term potentiation.

## Supporting Information

Figure S1 Acute BDNF stimulation does not induce a sustained increase in VGLUT protein levels after removal of the neurotrophin. (A–B) Cultured hippocampal neurons at DIV14 (A) and DIV7 (B) were incubated with 100 ng/ml BDNF for 4 hours in Neurobasal medium followed by a 14 h recovery period in culture conditioned medium. Total VGLUT1 (A) and VGLUT2 (B) protein levels were compared to control (without BDNF) expression, upon normalization with β-Tubulin levels. Quantification of ten different experiments, performed in independent preparations, is presented as mean percentage ±SEM compared to the control (unstimulated neurons). The differences obtained are not statistically significant, as determined by paired Student’s *t*-test with a confidence interval of 95%.
(TIF)

Figure S2 TrkB receptor inhibition has no effect on VGLUT2 expression. Cultured hippocampal neurons at DIV14 were incubated with a selective inhibitor of tyrosine protein kinase activity K252a (200 nM) and VGLUT2 protein levels were determined by western blot. Quantification of the indicated number of experiments, performed in independent preparations, is presented as mean percentage ±SEM compared to the control (unstimulated neurons). Statistical significance was determined by paired Student’s *t*-test with a confidence interval of 95%.
(TIF)
