# Supplementary figures and images for: BDNF Regulates the Expression and Distribution of Vesicular Glutamate Transporters in Cultured Hippocampal Neurons
Source: PLoS One. 2013 Jan 11;8(1):e53793. doi: 10.1371/journal.pone.0053793 (PMC3543267; doi:10.1371/journal.pone.0053793)

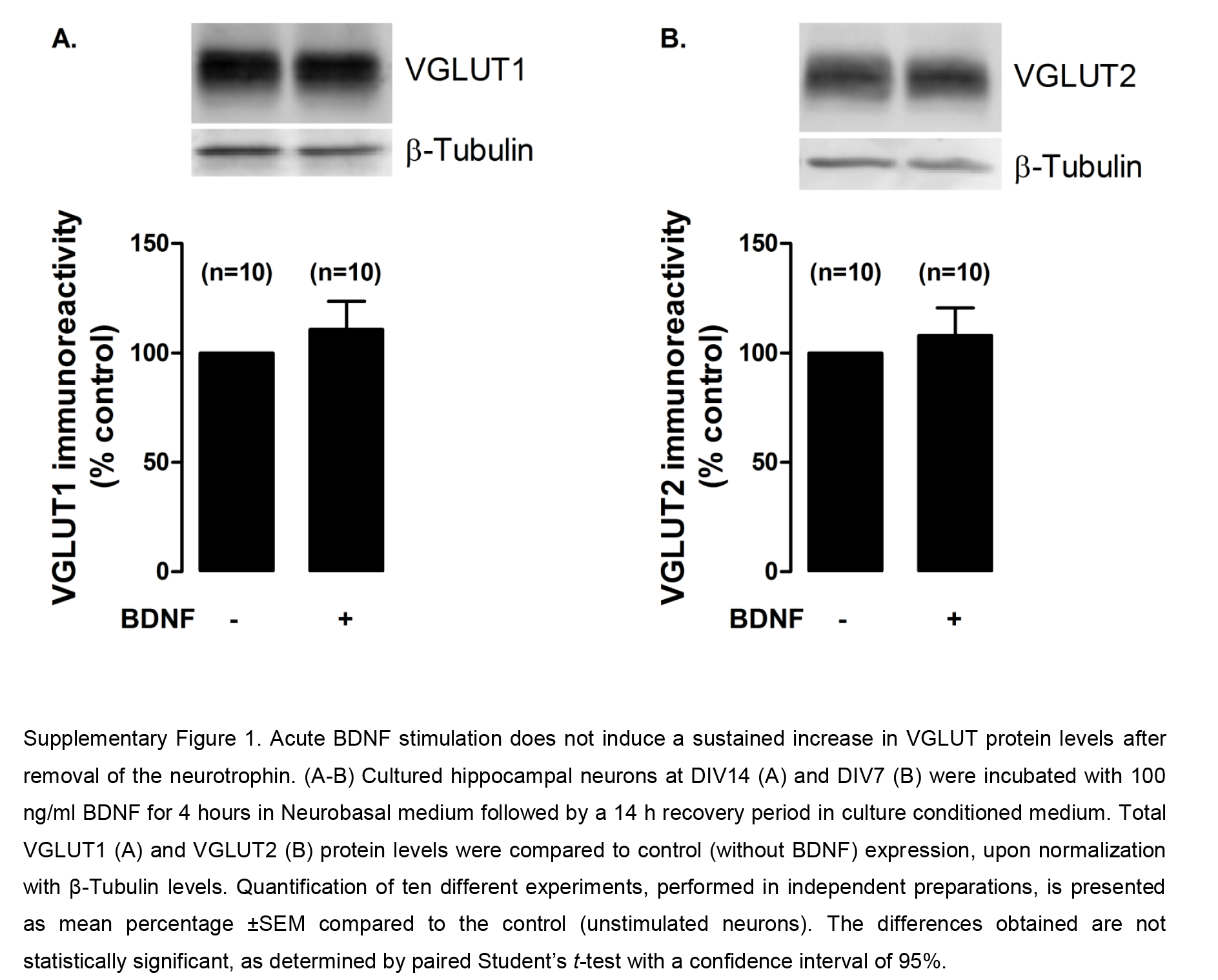

Supplement: Figure S1 — Acute BDNF stimulation does not induce a sustained increase in VGLUT protein levels after removal of the neurotrophin. (A–B) Cultured hippocampal neurons at DIV14 (A) and DIV7 (B) were incubated with 100 ng/ml BDNF for 4 hours in Neurobasal medium followed by a 14 h recovery period in culture conditioned medium. Total VGLUT1 (A) and VGLUT2 (B) protein levels were compared to control (without BDNF) expression, upon normalization with β-Tubulin levels. Quantification of ten different experiments, performed in independent preparations, is presented as mean percentage ±SEM compared to the control (unstimulated neurons). The differences obtained are not statistically significant, as determined by paired Student’s t-test with a confidence interval of 95%. (TIF) [file pone.0053793.s001.tif]

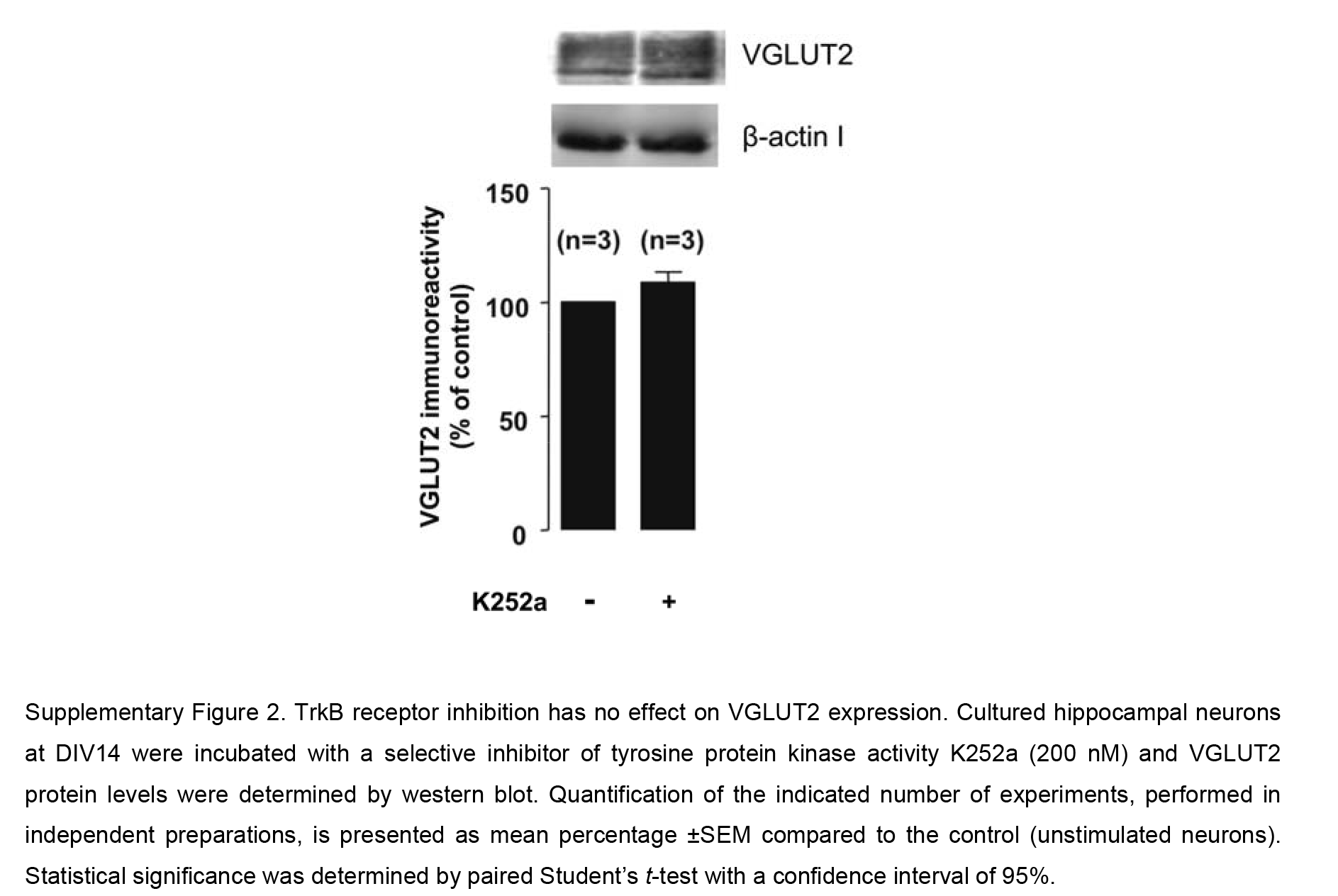

Supplement: Figure S2 — TrkB receptor inhibition has no effect on VGLUT2 expression. Cultured hippocampal neurons at DIV14 were incubated with a selective inhibitor of tyrosine protein kinase activity K252a (200 nM) and VGLUT2 protein levels were determined by western blot. Quantification of the indicated number of experiments, performed in independent preparations, is presented as mean percentage ±SEM compared to the control (unstimulated neurons). Statistical significance was determined by paired Student’s t-test with a confidence interval of 95%. (TIF) [file pone.0053793.s002.tif]
